# The Role of Social Power in Neural Responses to Others’ Pain

**DOI:** 10.3389/fpsyg.2019.02320

**Published:** 2019-10-15

**Authors:** Xueling Ma, Kai Wu, Entao Zhang

**Affiliations:** ^1^Institute of Cognition, Brain and Health, Henan University, Kaifeng, China; ^2^Institute of Psychology and Behavior, Henan University, Kaifeng, China

**Keywords:** social power, empathy, P1, ERP, pain

## Abstract

Recent evidence has demonstrated that empathic responses are modulated by social power. However, there is little consensus regarding how an observer’s social power can shape empathic responses. The present study used event-related potentials (ERPs) to explore the role of social power in empathic responses. Specifically, to induce the sense of power, we asked participants to recall a past situation in which they were in a position of power (high power prime) or a situation in which they were lacking power (low power prime). Afterward, we used ERPs to record the responses when participants were viewing pictures depicting other people in painful or non-painful situations. The results revealed that larger amplitudes in the earlier P2 and the later P3 components in response to painful stimuli than to non-painful stimuli. Besides, participants primed with high power only showed larger P1 amplitudes than participants primed with low power. The present study extended previous studies by showing that social power tends to enhance the early sensory processing of both painful and non-painful stimuli, instead of directly decreasing the level of empathic responses to others’ pain.

## Introduction

Empathy refers to the ability to share and understand the emotional states of others (Decety and Jackson, 2004). This ability is crucial for people’s successful social interaction with others. According to the “shared representations” account of empathy (De Vignemont and Singer, 2006), observing another person in a particular emotional state generates a similar emotional state in oneself. Consistent with this view, brain imaging studies have demonstrated that merely observing pain in others can activate brain regions mediating affective and somatosensory pain in the observer (Decety and Jackson, 2004; Jackson et al., 2005). This phenomenon is presently explained by assuming that empathic responses to others’ pain may occur automatically (Dimberg and Thunberg, 1998; Chartrand and Bargh, 1999; Dimberg et al., 2000; Han et al., 2008; Kramer et al., 2010).

However, recent several theories of emotions (Barrett, 2012; Mesquita and Boiger, 2014) proposed that emotions emerge from specific social interaction contexts. According to this view, each instance of any emotion is constructed by social interactions in which it takes place. For example, angry expressions are judged as a stronger signal of threat when they are shown by high-status people compared to low-status people (Ratcliff et al., 2012). It has to be pointed out that these theories do not deny that emotions are embodied, they just stress that emotions are situated in specific social contexts.

Consistent with this view, there are increasing evidence that empathic responses to others’ pain were also modulated by social factors, such as interpersonal relations (Singer et al., 2006; Beeney et al., 2011; Cui et al., 2015), the social status (Boksem et al., 2009; Guo et al., 2012; Varnum et al., 2015; Feng et al., 2016). The present study aims to examine the role of the observer’s social power in empathic responses to others’ pain.

Social power is a fundamental concept of social life and impacts a wide range of important and beneficial individual outcomes (Podolny, 2005). Power may constitute and change the social context in which emotions occur. In the psychological literature, social power refers to an individual’s relative ability to influence his or her partner’s outcomes by controlling resources and punishments (Keltner et al., 2003). Social power has been measured by assessing generalized sense of power as a personal disposition (e.g., Anderson and Galinsky, 2006; Anderson et al., 2012). In most past research, power was activated by asking participants to imagine themselves in or simulate the role of a manager or a subordinate (e.g., Guinote et al., 2002; Guinote, 2008) or via a mindset priming method, which asked participants to recall either a situation in which they possessed power over someone else or a situation in which someone else possessed power over them (Galinsky et al., 2003). Among those techniques, relative to other power manipulations, such as word search task, the recall priming task by Galinsky et al. (2003) has been shown to have far-reaching effects on a variety of behavioral outcomes, including ability to recognize facial emotional expressions (Galinsky et al., 2006) and to ignore peripheral information and focus on task-relevant details (Guinote, 2007a,b).

Major power theories assumed that social power leads to reduced processing of others’ emotions (Keltner et al., 2003; Russell and Fiske, 2010; Magee and Smith, 2013). Specifically, high-power individuals, because they control resources, tend not to attend to others’ emotions. Thus, high-power people show low empathic accuracy compared to low-power people (Keltner et al., 2003; Van Kleef et al., 2008). In line with this view, numerous studies have shown that people with high power are less accurate in recognizing others’ emotional expressions (Galinsky et al., 2006) or prosody (Uskul et al., 2016), and show lower levels of motor resonance than individuals with low power (Carr et al., 2014; Hogeveen et al., 2014).

In contrast, there is also conflicting evidence that individuals with a higher sense of power are associated with better facial emotion recognition or increased empathic accuracy (Schmid Mast et al., 2009; Côté et al., 2011). A recent meta-analysis (Hall et al., 2015) revealed the weak effect of power on emotion (averaged correlation = 0.07). One possible explanation for this is that the different power measurements or manipulations might require and affect different cognitive processing (Smith and Magee, 2015), thereby leading to different impacts on emotion. For example, the different aspects of power (feeling respect from others or the sense of controlling others) might modulate the power – empathy link. Recently, Magee and Galinsky (2008) argued that power is considered to be different from status, which refers to the relative level of respect and admiration one is conferred by others (Magee and Galinsky, 2008). Unfortunately, most of the previous researchers did not distinguish status from power when they assessed the impact of power on emotion. According to the widely accepted definition and manipulation method of power (Keltner et al., 2003), we argued that the controlling dimension of power is its core character. Thus, in the present study, we manipulated the social power by asking participants to recall and describe a particular incident in which they had power over another individual (high power prime) or someone else had power over them (low power prime).

Another limitation of previous studies is that empathic accuracy (the difference between the perceiver’s perception and the partner’s reported emotion) is usually used to test the effect of power on empathy. However, this behavioral method cannot assess the different stages of empathic responses to others’ emotions. In the present study, we used event-related potential (ERP), because of its excellent temporal resolution. The ERP technique is well-suited to assess the temporal dynamics of this study. ERPs can differentiate specific cognitive processes by linking them with neural components, depending on their activation time course and topography in brain areas. Also, ERP can provide critical temporal information for precise analysis of the timing of empathy.

Previous ERP studies have shown that earlier (N1 and P2) and later (P3) components were revealed when observing other people in painful or non-painful situations. The earlier components reflect the affective response of empathy for pain, while the later components involve the cognitive processing of empathy for pain (e.g., Pratto and John, 1991; Han et al., 2008; Ibáñez et al., 2011; Meng et al., 2012, 2013; Lyu et al., 2014). Specifically, previous studies have suggested that the N1 component is an expression of the early effects of the pain scene response, an automatic processing in the process of pain empathy, and an early automatic activation and sharing process of emotion. Previous studies have found that P2 is sensitive to negative stimuli, which reflects that negative stimuli receive more attention (Dowman, 2007; Chen et al., 2008; Yang et al., 2010; Fields and Kuperberg, 2012). Studies on pain empathy have consistently found that P2 is modulated by stimuli, being of larger amplitude to the painful than non-painful stimuli (Fan and Han, 2008). P3 reflects the evaluation and judgment process of the stimulus. Compared with N1, P3 illustrates the evaluation and control processing of pain empathy, which is a conscious evaluation of stimulus after automatic processing of perception and emotional cues. P3 is the top-down attention to pain cues in stimuli (Polich, 2007; Dufey et al., 2011).

In the present study, we used ERP to test whether individual power affects neural responses when viewing other people in painful or non-painful situations. Before participants received painful or non-painful pictures, we manipulated the social power by asking them to recall and describe a particular incident in which they had power over another individual (high power prime) or someone else had power over them (low power prime). In short, we hypothesized that power would modulate neural empathic responses to painful stimuli. Specific to the ERP component, we predicted that the empathy-related N1, P1, and P2 responses would be negatively correlated with power, such that those high power should show reduced neural empathic responses, but in P3, power would increase empathic responses.

## Materials and Methods

### Participants

A sample size of 40 undergraduate students participated in this study from Henan University and received financial compensation for their attendance in the study. The participants were alternately assigned to high power or low power condition. Besides, we discarded the data from two participants due to intensive head movements during EEG recording. Finally, 38 participants’ data were included (*M*_age_ = 21.4, *SD*_age_ = 1.23, 19 males). There were nineteen participants in each group. Based on self-report, no participant had a current or past history of neurological or psychiatric illness and all had normal or corrected-to-normal vision. This study was approved by the local Ethics Committee of Henan University, and all participants signed informed consent before the experiment.

### Apparatus and Stimuli

Electroencephalogram (EEG) was recorded from 32 scalp sites using tin electrodes mounted in an elastic cap (Brain Products, Brain Products GmbH, Gilching, Germany), arranged according to the International 10–20 System, with the reference on the right mastoid. EEG data were analyzed with the software Brain Vision Analyzer (Version 1.05; Brain Products, Munich, Germany).

**FIGURE 1:**
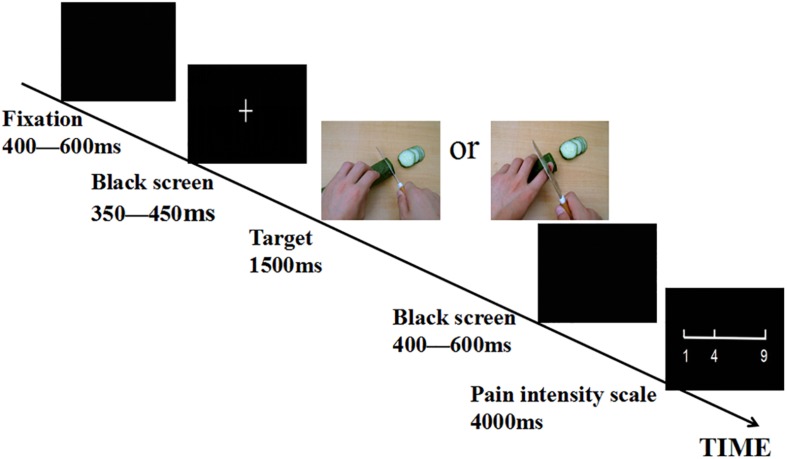
Illustration of the procedure.

The stimuli used in the experiment were pictures showing a person’s hands/feet in painful or non-painful situations (Figure 1), which have been used in previous ERP studies (Meng et al., 2012, 2013). All situations depicted familiar events that occasionally happen in everyday life. Image size 9 cm × 6.76 cm (width × height), definition, and luminance level of pictures were matched across priming conditions between painful and non-painful pictures (Meng et al., 2012, 2013). We opened the picture in Photoshop, select image – adjust – luminance level, and set the luminance level to 0. All pictures were presented on a black background (4.5°× 3.15°visual angle), with 100 pixels/in.

### Procedure

When the participants came to the laboratory, they first completed the agreeableness scale and the Interpersonal Reactivity Index. We used the 10-item agreeableness scale from the International Personality Item Pool (Goldberg, 1999). A sample item is “I make people feel at ease.” Responses were made using 5-point Likert scales (1 = very inaccurate, 5 = very accurate) (α = 0.75). Then we administered the 22-item interpersonal reactivity index (IRI) (Davis, 1983), including four dimensions, perspective taking, fantasy, empathy, and personal distress, we found no statistically significant difference in agreeableness and empathy between the high- and low-power participants (Table 1). Then primed with high or low power. Participants assigned to the high power condition were instructed to recall and write about an experience which they had power over another individual. Participants assigned to the low-power group were instructed to write about an experience in which another individual had power over them (Galinsky et al., 2003).

**TABLE 1:** Descriptive statistical values and differences between the scores of high and low power individuals.

	**High power**		**Low power**			
				
**Dimension**	***M***	***SD***	***M***	***SD***	***t***	***p***
Perspective taking	2.65	0.55	2.77	0.53	0.66	0.51
Fantasy	2.87	0.42	2.58	0.87	–1.30	0.20
Empathy	2.87	0.41	2.72	0.87	–0.91	0.37
Personal distress	1.74	0.50	1.99	0.69	1.24	0.17
Agreeableness	3.95	0.42	3.92	0.37	0.18	0.86

After completing power priming, participants were asked to take part in a sensory test in which they had observed painful or non-painful pictures. The experiment consisted of four formal experimental blocks of 60 trials each. The experiment started with 20 practice trials to familiarize participants with the task. As illustrated in Figure 1. In each trial, a fixation cross or point was presented on a black screen during a random duration between 400 and 600 ms. Subsequently, a blank screen was presented between 350 and 450 ms, then the painful or the non-painful pictures were displayed for 1500 ms, followed by a random duration between 400 and 600 ms followed by a blank screen, after which a 9-point pain intensity scale (1, no sensation; 4, pain threshold; 9, unbearable pain) appeared. Participants were asked to provide a rating by a button press with the right index or middle finger as quickly and accurately as possible. The scale remained onscreen until a response had been made, or for a 4 s maximum. The order of block conditions was counterbalanced across participants. The order of pictures was randomized within each block.

After the completion of the empathy test (the agreeableness scale and the IRI), participants were asked to respond to a 2-item power manipulation check (Kraus et al., 2011), indicating how much they agreed with each statement. The two items were “Now I feel I have a great sense of power” and “Now I feel my wishes don’t matter” (reverse scoring). Responses were made using 7-point Likert scales (1, “strongly disagree”; 7, “strongly agree”) (*r* = 0.89). The manipulation check confirmed that participants in the high power condition (*M* = 4.74, *SD* = 0.98) rated themselves as more powerful than those in the low power condition (*M* = 4.03, *SD* = 0.94), *t* (38) = −2.29, *p* = 0.028. Moreover, Following past research (Galinsky et al., 2003; Anderson and Galinsky, 2006), the effectiveness of the power manipulation was determined by having two condition-blind coders rate participants’ essays on content expressing high-power and low-power feelings (1, not at all; 7, very much) (*r* = 0.85), and therefore we combined the ratings of two coders to get a composite variable by averaging the ratings. As expected, participants in the high-power essays were rated as more powerful (*M* = 5.7, *SD* = 1.07) than participants in the low-power essays (*M* = 2.8, *SD* = 0.82), *t*(36) = −9.519, *p* < 0.001, *d* = 3.04. In addition, we calculated the correlation coefficient between our two manipulation checks (self-rating and coder’s rating), no significant correlation was found, *r* = 0.08, *p* = 0.687. We will discuss this point later in the discussion.

After the experiment, we asked participants whether they were aware of the link between the sensory test and the power induction, such as “Did you feel that there is something special about the experimental procedure?” “Did you know the purpose of the experiment?”, which confirmed that all participants were naive about the purpose of the study.

### EEG Recording and Analysis

To monitor eye movements and blinks, the vertical electrooculogram (VEOG) was recorded from electrodes placed on the supraorbital and infraorbital ridges of the right eye. EEG and EOG activity was amplified with a 0.01–100 Hz band-pass, and continuously sampled at 500 Hz. Impedance was below 5 kΩ for all recordings. Trials contaminated by blinks, eye movements, and excessive muscle activity were rejected offline (voltage exceeding ± 75 μV in any channel) before averaging. In sum, 10% of the trials were discarded from analysis.

The data were then re-referenced to the common average, after which the signal passed through a 0.01–30 Hz band-pass filter. Time windows of 200 ms before and 800 ms after the onset of the picture were segmented from EEG. Before seeing the data, we planned to deal with the data in terms of mean amplitude (see section “Results” in Supplementary Materials). After seeing the data, analyses were conducted over the peak amplitude of the N1 and P1 components and the mean amplitudes of the P2 and P3 component. Based on the topographical distribution of grand-averaged ERP activity and previous studies, different sets of electrodes for each component were chosen. The following 5 electrode sites F_z_, F3, F4, FC1, and FC2 were selected for the analysis of the N1 (110–160 ms); P3, P4, and Pz were selected for the analysis of the P1 (100–160 ms); F_z_, F3, F4, FC1, FC2, C3, C4, and C_z_ were selected for the analysis of the P2 (160–240 ms), P3, P4, Pz, CP1, CP2, O1, and O2 were selected for the analysis of the P3 (400–800 ms).

## Results

We used PP graph and histogram to check the normality. The results suggested that our data conformed to the normal distribution. We used the Levene test to check homoscedasticity, the results suggested that our data conformed to the homoscedasticity. Also, we used a discarding rule of ± 3 standard deviations for outliers. A mixed-model analysis of variance with Power condition (High/Low) as a between-subjects factor and Pain (Painful/Non-Painful) as a within-subject factor was performed for all selected electrodes sites for each component. To compensate violations of the sphericity assumption, we used Greenhouse-Geisser correction to correct the *P*-values. Bonferroni correction was used for multiple comparisons.

### Behavioral Performance

Reaction times (RT) and pain intensity ratings were calculated for each participant in each condition. The data were entered into a 2 (Power) × 2 (Pain) mixed model ANOVA with Power condition (High/Low) as a between-subjects factor and Pain (Painful picture/Non-Painful picture) as a within-subject factor. The analysis of RTs revealed a significant main effect of Picture, *F*(1,36) = 4.40, *P* < 0.005, $\eta_{p}^{2}$ = 0.11, Non-painful pictures (*M* = 596 ms, *SD* = 26) were recognized faster than painful pictures (*M* = 629 ms, *SD* = 31), the interaction of Power × Pain [*F*(1,36) = 0.437, *p* = 0.513, $\eta_{p}^{2}$ = 0.012] was not significant, the main effect of Power [*F*(1,36) = 0.222, *p* = 0.64, $\eta_{p}^{2}$ = 0.006] was not significant (see Table 2).

**TABLE 2:** Mean RTs and Pain intensity scale for each group.

**Power**	**Pain**	**RT (ms)**		**Pain intensity scale**	
			
		***M***	***SD***	***M***	***SD***
High power	Painful picture	637	175	5.94	1.19
	Non-painful picture	614	149	1.43	0.88
Low power	Painful picture	621	209	5.60	1.14
	Non-painful picture	577	175	1.17	0.18

The ANOVA for pain intensity showed a significant main effect of Picture, *F*(1,36) = 530.61, *p* < 0.0001, $\eta_{p}^{2}$ = 0.936, indicating that painful pictures (*M* = 5.77, *SD* = 0.19) were rated as significantly more painful than non-painful pictures (*M* = 1.298, *SD* = 0.103), interaction of Power × Pain intensity [*F*(1,36) = 0.043, *p* = 0.838, $\eta_{p}^{2}$ = 0.001] was not significant, the main effect of Power [*F*(1,36) = 1.645, *p* = 0.208, $\eta_{p}^{2}$ = 0.044] was not significant (see Table 2).

### ERP Results

#### N1

ANOVA on N1 revealed, the main effect of Power [*F*(1,36) = 0.328, *p* = 0.570, $\eta_{p}^{2}$ = 0.009], main effect of Pain [*F*(1,36) = 0.931, *p* = 0.341, $\eta_{p}^{2}$ = 0.025], and the interaction of Power × Pain [*F*(1,36) = 0.114, *p* = 0.738, $\eta_{p}^{2}$ = 0.003] were not significant. Meanwhile, a significant main effect of electrode site was observed, *F*(2,36) = 12.997, *p* < 0.0001, $\eta_{p}^{2}$ = 0.265, suggesting that largest amplitudes were elicited at the F4 (−3.69 μV) electrode sites.

#### P1

**FIGURE 2:**
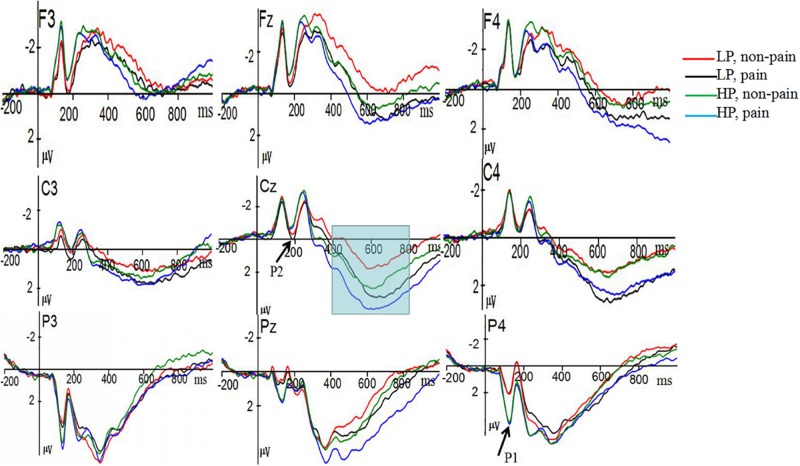
Grand average event-related potentials (ERP) elicited at electrodes F3, F_z_, F4, C3, C_z_, C4, P3, Pz, and P4 in response to painful and non-painful stimuli for high-power (HP) and low power (LP) participants.

ANOVA on P1 revealed, a marginal significant main effect of Power was observed [*F*(1,36) = 3.772, *p* = 0.06, $\eta_{p}^{2}$ = 0.095], indicating that participants in high power condition (*M* = 4.07 μV, *SE* = 0.53), elicited more positive P3 amplitudes than participants in low power condition (*M* = 2.61 μV, *SE* = 0.53). The main effect of Pain [*F*(1,36) = 0.947, *p* = 0.337, $\eta_{p}^{2}$ = 0.026], the interaction of Power × Pain [*F*(1,36) = 0.201, *p* = 0.657, $\eta_{p}^{2}$ = 0.006] were not significant. The main effect of electrode site was significant, *F*(2,36) = 18.429, *p* < 0.0001, $\eta_{p}^{2}$ = 0.339. Further analyses showed that largest amplitudes were elicited at the P3 (4.50 μV) electrode sites (see Figure 2).

#### P2

ANOVA on P2 revealed, the main effect of Power [*F*(1,36) = 1.453, *p* = 0.236, $\eta_{p}^{2}$ = 0.039] was not significant, we found that low-power participants (*M* = −0.791 μV, *SE* = 0.318) showed more positive amplitudes than high-power participants (*M* = −1.333 μV, *SE* = 0.318). We observed a significant main effect of Pain [*F*(1,36) = 5.725, *p* = 0.022, $\eta_{p}^{2}$ = 0.137]. Painful picture elicited a more negative P2 (*M* = −0.99 μV, *SE* = 0.23) than non-painful pictures (*M* = −1.13 μV, *SE* = 0.23). The interaction of Power × Pain [*F*(1,36) = 1.564, *p* = 0.219, $\eta_{p}^{2}$ = 0.042] did not reach significance. A significant main effect of electrode site was observed, *F*(3,36) = 11.112, *p* < 0.0001, $\eta_{p}^{2}$ = 0.236, suggesting that largest amplitudes were elicited at the F_z_ (−1.639 μV) electrode sites.

#### P3

ANOVA on P3 revealed, the main effect of Power [*F*(1,36) = 0.313, *p* = 0.579, $\eta_{p}^{2}$ = 0.009] and the interaction of Power × Pain [*F*(1,36) = 2.057, *p* = 0.16, $\eta_{p}^{2}$ = 0.054] were not significant, we found that high-power participants (*M* = 1.741 μV, *SE* = 0.46) showed more positive amplitudes than low-power participants (*M* = 1.38 μV, *SE* = 0.46). We found a significant main effect of Pain [*F*(1,36) = 7.308, *p* = 0.01, $\eta_{p}^{2}$ = 0.169], painful pictures elicited a significantly larger amplitude (*M* = 1.75 μV, *SE* = 0.30) than non-painful pictures (*M* = 1.37 μV, *SE* = 0.39). P3 amplitudes showed significant main effect at electrode size, *F*(2,36) = 20.951, *p* < 0.0001, $\eta_{p}^{2}$ = 0.236. Largest amplitudes were elicited at the CP2 (3.03 μV) electrode sites. None of the two-way, three-way, or four-way interaction reached significance (all *p*-values > 0.05).

To evaluate the strength of the empirical evidence, we also conducted a Bayesian analysis (Wagenmakers et al., 2017a, b). Bayesian analysis tests the strength of evidence between two theories (a null hypothesis theory and the proposed effect in the data), and its value ranges from 0 to infinity, with an increase in value indicating stronger support to reject the null hypothesis. The conventional cut-offs for Bayes factor sensitivity are 1/3 and 3, which means that any value outside of this range (less than 1/3 or greater than 3) provides strong evidence in support of the null hypothesis or the proposed effect in the data, respectively. Values between 1/3 and 3 are considered weak or “anecdotal” evidence. We found a Bayes factor of 1.417, which suggests that there is a difference between low-power and high-power individuals in RT. And a Bayes factor of 7.057e + 31 strongly supports the difference between low-power and high-power individuals in pain intensity. Consider the ERP results, a Bayes factor of 1.415 supports the difference between low-power and high-power individuals in pain intensity in P1, but there is anecdotal evidence for an effect of power on P1.

## Discussion

In some past studies, social power increased individuals’ empathic accuracy (e.g., Schmid Mast et al., 2009), in contrast, other studies have shown that social power decreased individuals’ empathic accuracy (e.g., Galinsky et al., 2006). In our study, we measured the ERP components of participants when they were viewing pictures depicting other people in painful or non-painful situations. The results revealed that larger amplitudes in the earlier P2 and the later P3 components in response to painful stimuli than to non-painful stimuli, suggesting that painful stimuli led to robust neural responses. In addition, participants primed with high power showed larger P1 amplitudes than participants primed with low power did. We will later discuss the implication of this finding.

Consistent with previous ERP studies about empathy for pain, the present study found that larger amplitudes in the earlier P2 and the later P3 components in response to painful stimuli than to non-painful stimuli (Han et al., 2008; Ibáñez et al., 2011; Meng et al., 2012; Lyu et al., 2014). The difference between painful and non-painful conditions was considered to be the participants’ P2 and P3 empathy effect. However, Power × Pain interaction absent in the P2 and P3 components, indicating that the social power of participants might not modulate empathic responses to others’ pain. The interaction of Power × Pain was statistically non-significant, which was not consistent with the result of Paulmann and Uskul (2017). In their study, the same power priming procedure was used to induce the sense of high or low power, and, then participants were asked to judge six different emotional voice. The authors found Emotion × Power interactions in P2 (200–250 ms) and P3 (450–850) components. To test our results, we used the Excel spreadsheet (Lakens, 2013) to compute the omega squared as well as 90% CI for eta-squared. For our Power × Pain interaction effect on P2, $\eta_{p}^{2}$ = 0.04, 90%CI for $\eta_{p}^{2}$ = 0,0.18, omega^2^p = 0.01. For Paulman & Uskul’s Emotion x Power interaction effect on P2, $\eta_{p}^{2}$ = 0.08, 90%CI for $\eta_{p}^{2}$ = 0.02, 0.12, omega^2^p = 0.06. However, as our research design was different from that of Paulmann and Uskul (2017), it is impossible to compare your partial eta^2^ with theirs. Yet, the results mentioned above would help researchers to get better understanding of these studies. In addition, the possible explanation for our non-significant interaction is that different emphatic test might require different cognitive processing, general emotional stimuli were used in a previous study, whereas the physical painful stimuli were used in our study. Because pain stimuli might be very salient or vital for all participants, regardless of whether they have high or low-power sense, these stimuli could not distinguish low-power participants from high-power participants. However, it has to be noted that our results are not mutually exclusive with the previous study. In contrast, future studies should focus on the effect of power on empathy in empathic tests about various emotions.

In addition, the most important result of this study was that participants primed with high power tended to show larger amplitudes than participants primed with low power did in the earlier P1 stage. However, the main effect of Pain and the Power × Pain interactions were not statistically significant in the P1 stage. The null effect of Pain in P1 suggested that P1 could not distinguish painful from non-painful events, and empathy effect did not occur in the P1 stage. The P1 component in visual areas has been related to the early sensory encoding of emotional stimuli. Some ERP studies have shown evidence for an enhanced P1 component for negative relative to neutral stimuli (for review see Vuilleumier, 2005). This finding suggested that in the initial stage of all the stimuli processing high-power participants are more sensitive to the stimuli than lower-power participants. In the late stages, both high and low-power participants show the same level-responses to pain stimuli, as these stimuli are too salient. In other words, social power enhanced individuals’ attention to the target goal. Our view is in line with the results of Côté et al. (2011), who argued that elevated power just enhanced goal focus rather than directly elevating or diminishing empathic accuracy. Recently, there was a work showing greater attunement of powerholders to their sensory states, for example, motor fluency (Woltin and Guinote, 2015; see also Guinote, 2017). Thus, together with work by Guinote (2017), our study at least demonstrated social power affected individuals’ sensory stage.

In line with this view, there is increasing evidence that social situations modulate emotional processing (e.g., Hogeveen et al., 2014; Uskul et al., 2016). However, it is unclear how the social situation affects emotional processing. In most previous studies, emphatic accuracy (EA) is usually used to test the effect of power on the emphatic response, and the correspondence between observer’s emotional judgment and target’s self-report is computed as the indicator of emphatic accuracy. In the present study, the effect of power on pain empathy was examined by using ERP, because of its excellent temporal resolution. The results have shown that participants primed with high power only showed larger P1 amplitudes than participants primed with low power did. In other words, there is a trend that social power as an important social situation enhanced individuals’ sensory processing.

According to situated emotions theories (Barrett, 2012; Mesquita and Boiger, 2014), social power affects emotional responses. These theories emphasize that emotions are situated in social contexts, rather than relative isolation. That is, emotions are closely tied to the interpersonal contexts in which they occur. This view has challenged the previous view of basic emotions proposed by Ekman (1992). The view of basic emotion assumes that at least some basic emotions are intrinsic and biological phenomena, which are linked with underlying physiological states and external facial expressions. According to this view, emotions take place at the interpersonal level, and are independent of the interpersonal contexts in which they take place. However, there is converging evidence that the processing of emotions can be modulated by social contexts in which they occur, such as the social status or power of the observer or the target (Guo et al., 2012; Varnum et al., 2015; Feng et al., 2016). These findings suggest that there are close links between social contexts and emotions, and social context should be taken into account in future emotional studies.

Moreover, the difference between the self and the blind coder’s rating in manipulation check would be considered in future studies. As initial manipulation check, participants were asked to indicate which they felt powerful, we found an unstandardized mean difference of 0.71 unit of our power manipulation with a 95% CI for unstandardized μ (0.10, 1.32), Cohen’s *d* ≈ 0.71. However, using the same blind-coder-based manipulation check as in previous studies, we observed an unstandardized mean difference of 2.8 units of the 7-point Lickert scale, 95% CI for the unstandardized μ(2.28,3.33). In addition, using ESCI (Cumming, 2012) to conduct a small-scale meta-analysis of the effect of the autobiographical power manipulation on the coder’s ratings including six studies (Anderson and Galinsky, 2006, Studies 2 and 4; Galinsky et al., 2003, Studies 2 and 3; Galinsky et al., 2006, Study 1; Yang et al., 2015, Study 1), it has been found an average unstandardized mean difference of 3.3 units of the 7-point Lickert scale, 95% CI for the unstandardized μ(2.93,3.67). Thus, although the meta-analysis was limited because of its small scale, the effect size of our manipulation on the manipulation check is not significantly different than those of the meta-analysis. In addition, there was no significant correlation between two manipulation checks. One possible explanation was that self-rating by participants might be affected by both participants’ internal feelings and subjective standards, whereas the coder’s rating might control participants’ subjective standards. However, this explanation should be taken with caution, further studies would be needed to exam this difference.

Lastly, our current study did not find Power × Pain interactions, several potential limitations must be noted. First, the lack of a power-control condition is our limitation, adding a power-control condition would be considered in our future study. Secondly, alternate assignment of each participant in experimental conditions could affect our results. Finally, our effect was not statistically significant presumably because of an underpowered research design.

## Conclusion

Taken together, the present results showed that power tends to enhance sensory processing of both painful and non-painful stimuli, instead of decreasing the level of empathic responses to others’ pain.

## Ethics Statement

This study was approved by the local Ethics Committee of Henan University, and informed written consent was signed from all participants before the experiment.

## Author Contributions

EZ and XM designed the study and drafted the manuscript. XM and KW performed the study. EZ, KW, and XM analyzed the data. EZ reviewed the manuscript.

## Conflict of Interest

The authors declare that the research was conducted in the absence of any commercial or financial relationships that could be construed as a potential conflict of interest.
