# Supplementary material for: The Role of Social Power in Neural Responses to Others’ Pain
Source: Front Psychol. 2019 Oct 15;10:2320. doi: 10.3389/fpsyg.2019.02320 (PMC6804528; doi:10.3389/fpsyg.2019.02320)
Supplement: Supplementary file 1 [file Data_Sheet_1.doc]

Analyses were conducted over the mean amplitude of the N1 and P1 components. On the basis of the topographical distribution of grand-averaged ERP activity and previous studies, different sets of electrodes for each component were chosen. The following 5 electrode sites Fz, F3, F4, FC1, FC2 were selected for the analysis of the N1 (80-150 ms); P3, P4, Pz, O1, O2 were selected for the analysis of the P1 (100-160 ms).

N1：

ANOVAs on N1 revealed, the main effect of Power (F (1, 36) = 0.808, p = 0.375, $\eta_{p}^{2}$ = 0.022), main effect of Picture (F (1, 36) = 1.605, p = 0.213, $\eta_{p}^{2}$= 0.043), and the interaction of Power ×Picture (F (1, 36) = 0.554, p = 0.462, $\eta_{p}^{2}$= 0.015) were not significant. Meanwhile, a significant main effect of electrode site was observed, F (2, 36)= 6.345, p < 0.001, $\eta_{p}^{2}$ = 0.150, suggesting that largest amplitudes were elicited at the F3 (-1.45µV) electrode sites .

P1:

ANOVAs on P1 revealed, the main effect of Power (F (1, 36) = 1.577, p = 0.217, $\eta_{p}^{2}$ = 0.042), main effect of Picture (F (1, 36) = 0.124, p = 0.727, $\eta_{p}^{2}$= 0.003), the interaction of Power ×Picture (F (1, 36) = 0.036, p = 0.850, $\eta_{p}^{2}$= 0.001) were not significant. The main effect of electrode site was significant, F (2, 36) = 55.009, p < 0.0001, $\eta_{p}^{2}$= 0.604. Further analyses showed that largest amplitudes were elicited at the O1 (6.06µV) electrode sites.
